# Between-hospital variations in 3-year survival among patients with newly diagnosed gastric, colorectal, and lung cancer

**DOI:** 10.1038/s41598-022-11225-5

**Published:** 2022-05-03

**Authors:** Toshitaka Morishima, Sumiyo Okawa, Shihoko Koyama, Kayo Nakata, Takahiro Tabuchi, Isao Miyashiro

**Affiliations:** Cancer Control Center, Osaka International Cancer Institute, 3-1-69 Otemae, Chuo-ku, Osaka, 541-8567 Japan

**Keywords:** Health care, Oncology

## Abstract

Due to increases in cancer survivability, quality assessments of cancer care must include long-term outcomes. This multicenter retrospective cohort study evaluated between-hospital variations in the 3-year survival rates of patients with gastric, colorectal, and lung cancer irrespective of treatment modality. We linked cancer registry data and administrative data from patients aged 18–99 years who were diagnosed with gastric, colorectal, or lung cancer between 2013 and 2015 in Osaka Prefecture, Japan. The 3-year survival rates were adjusted for potential prognostic factors using multilevel logistic regression models. Between-hospital variations were visually evaluated using funnel plots. We analyzed 10,296 gastric cancer patients from 30 hospitals, 9276 colorectal cancer patients from 30 hospitals, and 7978 lung cancer patients from 28 hospitals. The 3-year survival rate was 70.2%, 75.2%, and 45.0% for gastric, colorectal, and lung cancer, respectively. In the funnel plots, the adjusted survival rates of gastric and colorectal cancer for all hospitals lay between the lower and upper control limits of two standard deviations of the average survival rates. However, the adjusted survival rates of lung cancer for four hospitals lay below the lower limit while that for two hospitals lay above the upper limit. Older age, men, advanced cancer stage, comorbidities, functional disability, emergency admission, current/ex-smokers, and underweight were independently associated with poorer survival. In conclusion, there were between-hospital variations in 3-year survival for lung cancer even after adjusting for case mix. Quality improvement initiatives may be needed to raise the consistency of care.

## Introduction

Countries with universal health coverage should, in theory, have consistently high standards of treatment with limited variations in quality across healthcare institutions^1^. To achieve this goal, quality assessments have been conducted to identify and minimize any extraneous heterogeneity in clinical practice^2,3^. Numerous studies have reported wide disparities in clinical care processes for cancer patients at the hospital level^4–8^, which may lead to variations in survival rates. We therefore hypothesized that survival outcomes after cancer diagnosis vary among hospitals. If this hypothesis is true, there may be considerable scope for improving the survivability of cancer patients^9^.

Comparisons of hospital performance based on unadjusted outcomes are problematic due to inherent differences in patient case mix. Prognostic factors such as patient age, comorbidities, and disease severity can influence overall survival in cancer populations^10^, and these factors should be controlled to enable robust comparisons that account for case mix differences^11^. Although cancer registries provide a wealth of information on tumor incidence, characteristics, and outcomes, their datasets are generally inadequate for outcome comparisons^12^. This is because cancer registry data frequently lack important clinical information required for risk adjustments. In contrast, administrative claims data contain detailed clinical information, but lack records on tumor characteristics and outcomes. The linkage of these two data types could therefore provide a more comprehensive dataset for analyses. In Japan, most acute care hospitals are reimbursed under the Diagnosis Procedure Combination (DPC) system, and the generated data are widely used for epidemiological research^13^. Cancer registry data and administrative data have been linked to allow for adjustments in case mix among Japanese cancer patients^14^. Previous studies have also shown that such linked data can be used to identify prognostic factors of cancer^15–20^.

The current literature on hospital variations in survival in the oncology setting is usually surgery-specific or focused on short-term mortality (e.g., 30-day postoperative mortality)^21–30^. Within the increasing culture of accountability in healthcare, short-term mortality will understandably remain one of the more important metrics for the surgical field. However, we consider it reasonable to include both surgical and non-surgical patients in analyses to appraise a hospital as a whole rather than separating patients according to their treatment modalities. This is because the decisions to perform resections are complex, and are based on the needs of each patient and the case selection of each surgeon. In addition, the mortality rates soon after surgery are low, making it unlikely that postoperative mortality would be sufficiently sensitive to detect significant differences among hospitals^29,31,32^. In contrast, survival metrics spanning a year or more at the hospital level are important for cancer patients and policymakers because survival duration is a major determinant of cancer care quality^33^.

Using a database that linked cancer registry data and administrative data, this study aimed to investigate between-hospital variations in 3-year survival rates for cancer patients in Japan irrespective of treatment modality, as well as to elucidate patient-level prognostic factors that affect survival.

## Methods

### Data sources

We performed a multicenter retrospective cohort study of 31 cancer care hospitals in Osaka Prefecture, Japan. These institutions were designated as cancer care hospitals by the national or prefectural government based on certain functional criteria for providing cancer treatment. The study database was produced through the linkage of hospital-based cancer registry data, prefectural cancer registry data, and administrative data. The data were obtained with support from the Council for Coordination of Designated Cancer Care Hospitals in Osaka^15–20^.

Hospital-based cancer registry data were collected from the cancer care hospitals. In addition to patient demographic information (age and sex), these data also included tumor information such as cancer diagnoses with topographical and morphological codes from the International Classification of Diseases for Oncology, Third Edition (ICD-O-3); cancer stage and tumor-node-metastasis classifications at diagnosis according to the Seventh Edition of the Union for International Cancer Control (UICC) staging system; and dates of cancer diagnoses. These hospital-based cancer registry data were linked to DPC administrative data for episodes of hospital care. The DPC data included inpatient and outpatient administrative claims for the provision of care, as well as the following discharge abstracts for inpatient episodes: dates of admission and discharge, patient characteristics (e.g., body height and weight), and the primary diagnosis and pre-existing comorbidities on admission according to International Classification of Diseases, Tenth Edition (ICD-10) codes^13^. Additionally, data from Osaka Cancer Registry, which collects and curates information on cancer incidence and outcomes in Osaka Prefecture residents, were also linked. These registry data contained the dates of cancer diagnoses and vital status information (verified through death certificates and official resident registries).

The three data sources were linked at the patient level and the completeness of record linkage was estimated to be 98%^14^. The 31 participating hospitals treated approximately 50% of all patients with newly diagnosed cancer in the study region. An anonymized dataset was extracted from the linked database for this study.

### Selection criteria

**Figure 1:**
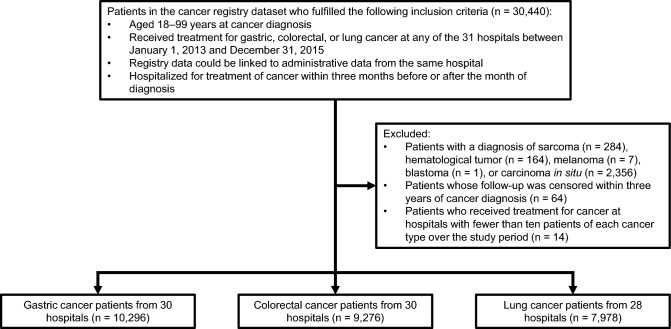
Flow diagram of study population selection.

The study population was selected using the hospital-based cancer registry dataset. The selection process is presented in Fig. 1. We first identified 30,440 patients (a) aged 18–99 years at cancer diagnosis; (b) who received treatment for gastric, colorectal, or lung cancer (designated the index cancer) at any of the 31 hospitals between January 1, 2013 and December 31, 2015; (c) whose registry data could be linked to administrative data from the same hospital; and (d) who were hospitalized for treatment of the index cancer within 3 months before or after the month of diagnosis. Treatment of the index cancer was defined as the first course of cancer-specific treatment, including best supportive care. We assigned each patient and his/her outcome to the hospital that provided this initial treatment even if he/she subsequently visited other hospitals for continued cancer care. We selected the three target cancer types due to their relatively high incidence within the study region, and identified them using ICD-O-3 topographical codes (C16.x for gastric cancer, C18.x–C20.x for colorectal cancer, and C33.x–C34.x for lung cancer). If an individual patient received multiple diagnoses of the same cancer type, we used the information from the earliest diagnosis. Patients were excluded if they had a diagnosis of sarcoma (ICD-O-3 morphological codes: 8800–8936, 8990–8991, 9020, 9040–9044, 9120–9133, 9150, 9170, and 9180–9251; n = 284), hematological tumor (9590–9989; n = 164), melanoma (8720–8790; n = 7), blastoma (8970–8974; n = 1), or carcinoma in situ (n = 2356); or if the follow-up of their vital status was censored within 3 years of cancer diagnosis (n = 64). We also excluded patients from hospitals with fewer than ten patients of each cancer type over the 3-year study period to ensure sufficient caseloads and to avoid extreme values. This led to the exclusion of 14 lung cancer patients from two hospitals. The final study population comprised 27,550 patients from 30 hospitals. One of the target hospitals had no eligible patients.

### Potential prognostic factors

From the hospital-based cancer registry data, we obtained the following pretreatment demographic and tumor factors that can potentially influence outcomes: age (18–64, 65–69, 70–74, 75–79, and 80–99 years), sex, and UICC stage (I, II, III, IV, and unknown) at cancer diagnosis. The UICC pathological classification system was primarily used to determine cancer stage, but the clinical classification system was used for cases where surgical resections were not performed. For colorectal cancer, tumor localization was also included as a prognostic factor, and was grouped into right-sided colon cancer (ICD-O-3 topographical codes: C18.0–C18.5) and left-sided colorectal cancer (C18.6–C18.7, C19.9, C20.9) due to differences in tumor biology and prognoses between the anatomical subsites^34^. Patients with an ICD-O-3 topographical code of C18.8 or C18.9 (unspecified localization of colon cancer) in the hospital-based cancer registry data were manually classified to the appropriate subsite according to their primary diagnoses (ICD-10 codes) in the administrative data. For lung cancer, tumor histology was also included as a prognostic factor, and was classified into small cell lung cancer (ICD-O-3 morphological codes: 8041–8045) and non-small cell lung cancer (all other ICD-O-3 morphological codes)^35^.

For each patient identified in the hospital-based cancer registry data, we searched all inpatient episodes in the administrative data to identify the first cancer-specific hospitalization within 3 months before or after the month of cancer diagnosis. The following pretreatment demographic factors were derived from the administrative data: pre-existing comorbidities on admission, activities of daily living (ADL) on admission, type of admission (emergency or elective), smoking status (never smoker, current or ex-smoker, or unknown), and body mass index (BMI). The Quan adaptation of the Charlson Comorbidity Index (CCI) based on ICD-10 codes was used to measure patient comorbidities (excluding metastasis), and patients were grouped into three categories: no comorbidity (CCI score: 0), moderate comorbidities (1–2), and severe comorbidities (≥ 3)^36,37^. This procedure has been previously described in detail^14^. Higher CCI scores indicate a higher risk of death. The Barthel index was used to measure performance in ADL because of its association with cancer survival^15,16^. This index uses a scale of 0–100 with higher scores indicating better functional status, and patients were grouped into four categories: no disability (score: 100), moderate disability (60–95), severe disability (0–55), and unknown^15^. Emergency admission was defined as hospitalization due to critical conditions such as bleeding, perforation, shock, and organ failure; all other admissions were considered elective. Emergency admission was included as it has been reported to be a stage-independent poor prognostic factor^38,39^. Finally, BMI was calculated as weight (kg)/height (m^2^), and patients were grouped into five categories: underweight (BMI: < 18.5), normal weight (18.5–24.9), overweight (25.0–29.9), obese (≥ 30), and unknown^16^.

### Statistical analysis

The study outcome was patient survival for 3 years or more. We defined overall survival duration as the period from the date of cancer diagnosis until the date of all-cause mortality. The 3-year survival rate was expressed as a proportion (percentage) of the total caseload. We constructed multilevel logistic regression models with hospital as a random effect to determine the variables associated with the probability of 3-year survival. The outcome variable had a binary value (survival or non-survival). The potential prognostic factors described above were all entered into the statistical models as explanatory variables. Missing data values (i.e., “unknown” categories) for cancer stage, ADL, smoking status, and BMI were imputed using five datasets from multiple imputation models that incorporated all explanatory and outcome variables^40^. We assumed that the missing data were missing at random. This assumption was plausible because of the wide range of variables included in the multiple imputation models. As a sensitivity analysis, the models used to investigate the associations of the potential prognostic factors with overall survival were built using a dataset restricted to cases with complete data (i.e., exclusion of patients with missing data values for cancer stage, ADL, smoking status, or BMI). The statistical analyses were separately performed for gastric, colorectal, and lung cancer patients using SAS 9.4 (SAS Institute, Cary, NC). Two-tailed *P* values below 0.05 were considered statistically significant.

### Funnel plots

Institutional variations in 3-year survival rates were examined using funnel plots, which are designed to detect variations through simple visual inspection^41^. Greater variations in healthcare quality metrics can arise by chance in hospitals with smaller caseloads due to the between-hospital variations in cumulative caseload during the 3-year study period. Individual hospitals were plotted according to the outcome measure (Y-axis) and caseload during the study period (X-axis).

Three statistical models were fitted to create a set of graphs for each cancer type in order to assess the performance of the risk adjustment process: one for unadjusted outcomes and two for risk-adjusted outcomes. The first risk adjustment was performed using multilevel logistic regression models that included explanatory variables extracted only from the hospital-based cancer registry data (i.e., age, sex, stage, tumor localization for colorectal cancer, and histology for lung cancer). The outcomes produced by these models were termed “partially adjusted survival rates”. The second risk adjustment was performed using models that also included explanatory variables extracted from the administrative data (i.e., CCI, ADL, type of admission, smoking status, and BMI) in addition to the explanatory variables from the cancer registry data. The outcomes produced by these models were termed “fully adjusted survival rates”. To obtain the adjusted survival rates for each hospital, the observed number (O) of patients who survived for 3 years or more was divided by the expected number (E) to calculate an O/E ratio that was multiplied by the average survival rate (i.e., the crude number of 3-year survivors divided by the crude number of selected patients) of all hospitals (shown as the horizontal line in each graph)^11^. Each hospital’s expected number of survivors was calculated by summing its patients’ predicted probabilities of surviving given their individual values in the explanatory variables, and averaging over the random effect. Poisson control limits for a given level of significance were also drawn around the horizontal line. We used two-sided significance levels of 0.05 and 0.002, and the resulting 95% and 99.8% control limits represented approximately 2 (inner) and 3 (outer) standard deviations (SDs), respectively, on either side of the horizontal line. Hospitals with survival rates below the lower control limit or above the upper control limit of 2 SDs were regarded as potential outliers^23–29^. Adjusted survival rates below the lower control limit of 3 SDs and 2 SDs were regarded as “alarm” and “alert” signals, respectively^41^.

The distribution of cancer stage varied substantially among the hospitals. Preliminary analyses showed that the proportion of stage I cancer ranged from 44.4 to 74.4% in gastric cancer, 16.5% to 38.9% in colorectal cancer, and 0.0% to 48.3% in lung cancer. These variations can cause misleading comparisons of survival between hospitals because stage is a strong prognostic factor. Therefore, we conducted subgroup analyses for patients with stage I cancer. In these analyses, we excluded patients from hospitals with fewer than ten patients of each cancer type over the study period to ensure sufficient caseloads. This led to the exclusion of 17 stage I lung cancer patients from six hospitals.

### Ethical approval

The study was conducted in accordance with the relevant guidelines and regulations of the ethical principles for medical research involving human subjects, as stated by the Declaration of Helsinki. Ethical approval was granted by the Institutional Review Board of Osaka International Cancer Institute (Approval number: 1707105108). The Institutional Review Board of Osaka International Cancer Institute waived the need for informed consent in accordance with the Japanese government’s Ethical Guidelines for Medical and Health Research Involving Human Subjects, which allow for the opt-out approach for the secondary use of existing data.

## Results

The study population comprised 10,296 gastric cancer patients from 30 hospitals, 9276 colorectal cancer patients from 30 hospitals, and 7978 lung cancer patients from 28 hospitals (Fig. 1). Table 1 shows the distribution of pretreatment demographic and tumor characteristics. The unadjusted 3-year survival rate across the study hospitals was 70.2%, 75.2%, 45.0% for gastric, colorectal, and lung cancer, respectively. Among the age groups, patients aged 18–64 years formed the highest proportion of gastric and colorectal cancer cases, whereas patients aged 70–74 years formed the highest proportion of lung cancer cases. A high proportion of gastric and colorectal cancers were diagnosed at UICC stage I, whereas lung cancer presented more frequently at stage IV. Regardless of cancer type, the majority of patients had no comorbidity or functional disability, and had first received treatment in an elective admission. Current or ex-smokers were more prevalent among the gastric and lung cancer patients than never smokers, but never smokers were more prevalent among the colorectal cancer patients. Approximately two thirds of all patients had a BMI of 18.5–24.9 (normal weight). Proportions of the “unknown” categories for cancer stage, ADL, smoking status, and BMI ranged from 0.1 to 2.9%.

**Table 1:** Pretreatment demographic and tumor characteristics of patients with gastric, colorectal, and lung cancer.

	Gastric cancer (n = 10,296)	Colorectal cancer (n = 9276)	Lung cancer (n = 7978)
**Vital status**			
Survived 3 years or more	7226 (70.2%)	6971 (75.2%)	3589 (45.0%)
**Age (years)**			
18–64	2335 (22.7%)	2522 (27.2%)	1799 (22.5%)
65–69	1877 (18.2%)	1661 (17.9%)	1570 (19.7%)
70–74	2244 (21.8%)	1817 (19.6%)	1872 (23.5%)
75–79	2017 (19.6%)	1640 (17.7%)	1523 (19.1%)
80–99	1823 (17.7%)	1636 (17.6%)	1214 (15.2%)
**Sex**			
Male	7266 (70.6%)	5277 (56.9%)	5388 (67.5%)
**Stage**			
I	6400 (62.2%)	2536 (27.3%)	2565 (32.2%)
II	919 (8.9%)	2459 (26.5%)	742 (9.3%)
III	1104 (10.7%)	2529 (27.3%)	1568 (19.7%)
IV	1795 (17.4%)	1704 (18.4%)	3022 (37.9%)
Unknown	78 (0.8%)	48 (0.5%)	81 (1.0%)
**Tumor localization**			
Right-sided	‒	3150 (34.0%)	‒
**Histology**			
Small cell lung cancer	‒	‒	874 (11.0%)
**Comorbidity**			
No comorbidity	7726 (75.0%)	6627 (71.4%)	5263 (66.0%)
Moderate comorbidities	2235 (21.7%)	2324 (25.1%)	2323 (29.1%)
Severe comorbidities	335 (3.3%)	325 (3.5%)	392 (4.9%)
**ADL**			
No disability	9096 (88.3%)	7949 (85.7%)	6656 (83.4%)
Moderate disability	698 (6.8%)	624 (6.7%)	729 (9.1%)
Severe disability	487 (4.7%)	681 (7.3%)	580 (7.3%)
Unknown	15 (0.1%)	22 (0.2%)	13 (0.2%)
**Type of admission**			
Emergency	854 (8.3%)	1372 (14.8%)	702 (8.8%)
**Smoking status**			
Never smoker	4574 (44.4%)	4941 (53.3%)	2083 (26.1%)
Current or ex-smoker	5447 (52.9%)	4069 (43.9%)	5744 (72.0%)
Unknown	275 (2.7%)	266 (2.9%)	151 (1.9%)
**Body mass index**			
< 18.5	1223 (11.9%)	1158 (12.5%)	1089 (13.7%)
18.5–24.9	6816 (66.2%)	5950 (64.1%)	5180 (64.9%)
25.0–29.9	1893 (18.4%)	1769 (19.1%)	1390 (17.4%)
≥ 30.0	228 (2.2%)	260 (2.8%)	171 (2.1%)
Unknown	136 (1.3%)	139 (1.5%)	148 (1.9%)

*ADL* activities of daily living.

### Factors associated with 3-year survival

Table 2 summarizes the results of the multilevel logistic regression analyses examining the associations of the potential prognostic factors with 3-year overall survival after multiple imputations. The odds of survival were significantly lower for older age, men, and more advanced stages for all three cancer types. The adjusted odds ratios for these three variables in colorectal cancer were closer to 1.00 than the other two cancer types. Localization of colorectal cancer was not significantly associated with survival. Small cell lung cancer generated significantly lower odds than non-small cell lung cancer. Moderate and severe comorbidities were significantly associated with lower odds than no comorbidity for all cancer types, with the exception of moderate comorbidities in lung cancer. Functional disability in ADL was associated with significantly lower odds when compared with no disability. Emergency admission was also an independent risk factor of poorer survival. Current or ex-smokers with colorectal or lung cancer were significantly less likely to survive than never smokers. The odds of survival were lower for patients with a BMI of < 18.5 than for those with a BMI of 18.5–24.9 in all three cancer types, whereas the odds were higher for patients with a BMI of 25.0–29.9 in gastric and colorectal cancer. A BMI of ≥ 30 was not significantly associated with survival. In the sensitivity analysis, we repeated the multilevel logistic regression analyses using complete-case data (Supplementary Table S1). These results were similar to those of the main analyses using data from the multiple imputation models.

**Table 2:** Association of prognostic factors with 3-year overall survival in patients with gastric, colorectal, and lung cancer using data from multiple imputation models.

	Gastric cancer (n = 10,296)		Colorectal cancer (n = 9276)		Lung cancer (n = 7978)	
	Adjusted odds ratio	*P* value	Adjusted odds ratio	*P* value	Adjusted odds ratio	*P* value
**Age (years; Ref = 18–64)**						
65–69	0.83 (0.68–1.02)	0.077	0.92 (0.76–1.11)	0.393	0.69 (0.58–0.83)	< 0.001
70–74	0.62 (0.51–0.76)	< 0.001	0.79 (0.66–0.95)	0.014	0.55 (0.46–0.66)	< 0.001
75–79	0.43 (0.35–0.52)	< 0.001	0.50 (0.41–0.60)	< 0.001	0.33 (0.28–0.40)	< 0.001
80–99	0.25 (0.20–0.30)	< 0.001	0.32 (0.26–0.38)	< 0.001	0.18 (0.14–0.22)	< 0.001
**Sex (Ref = female)**						
Male	0.69 (0.59–0.80)	< 0.001	0.78 (0.68–0.90)	< 0.001	0.53 (0.46–0.62)	< 0.001
**Stage (Ref = I)**						
II	0.35 (0.29–0.42)	< 0.001	0.93 (0.77–1.12)	0.443	0.32 (0.26–0.38)	< 0.001
III	0.11 (0.09–0.13)	< 0.001	0.43 (0.36–0.52)	< 0.001	0.12 (0.10–0.14)	< 0.001
IV	0.01 (0.01–0.01)	< 0.001	0.04 (0.03–0.05)	< 0.001	0.03 (0.03–0.04)	< 0.001
**Tumor localization (Ref = left-sided)**						
Right-sided	‒		0.95 (0.84–1.08)	0.439	‒	
**Histology (Ref = NSCLC)**						
SCLC	‒		‒		0.40 (0.32–0.50)	< 0.001
**Comorbidity (Ref = no comorbidity)**						
Moderate comorbidities	0.64 (0.55–0.74)	< 0.001	0.62 (0.55–0.71)	< 0.001	0.88 (0.76–1.003)	0.056
Severe comorbidities	0.32 (0.24–0.43)	< 0.001	0.35 (0.26–0.45)	< 0.001	0.58 (0.44–0.77)	< 0.001
**ADL (Ref = no disability)**						
Moderate disability	0.49 (0.39–0.61)	< 0.001	0.47 (0.38–0.58)	< 0.001	0.51 (0.40–0.64)	< 0.001
Severe disability	0.20 (0.15–0.26)	< 0.001	0.30 (0.25–0.37)	< 0.001	0.19 (0.13–0.28)	< 0.001
**Type of admission (Ref = elective)**						
Emergency	0.66 (0.52–0.83)	< 0.001	0.66 (0.56–0.77)	< 0.001	0.48 (0.34–0.66)	< 0.001
**Smoking status (Ref = never smoker)**						
Current or ex-smoker	0.92 (0.80–1.05)	0.225	0.85 (0.74–0.98)	0.027	0.50 (0.42–0.59)	< 0.001
**Body mass index (Ref = 18.5–24.9)**						
< 18.5	0.56 (0.47–0.67)	< 0.001	0.64 (0.54–0.76)	< 0.001	0.55 (0.46–0.67)	< 0.001
25.0–29.9	1.23 (1.04–1.45)	0.014	1.37 (1.16–1.61)	< 0.001	1.14 (0.98–1.34)	0.099
≥ 30.0	1.54 (0.99–2.40)	0.057	1.22 (0.84–1.79)	0.300	1.01 (0.67–1.52)	0.959

*ADL* activities of daily living, *NSCLC* non-small cell lung cancer, *Ref* reference, *SCLC* small cell lung cancer.

### Between-hospital variations in 3-year survival

**Figure 2:**
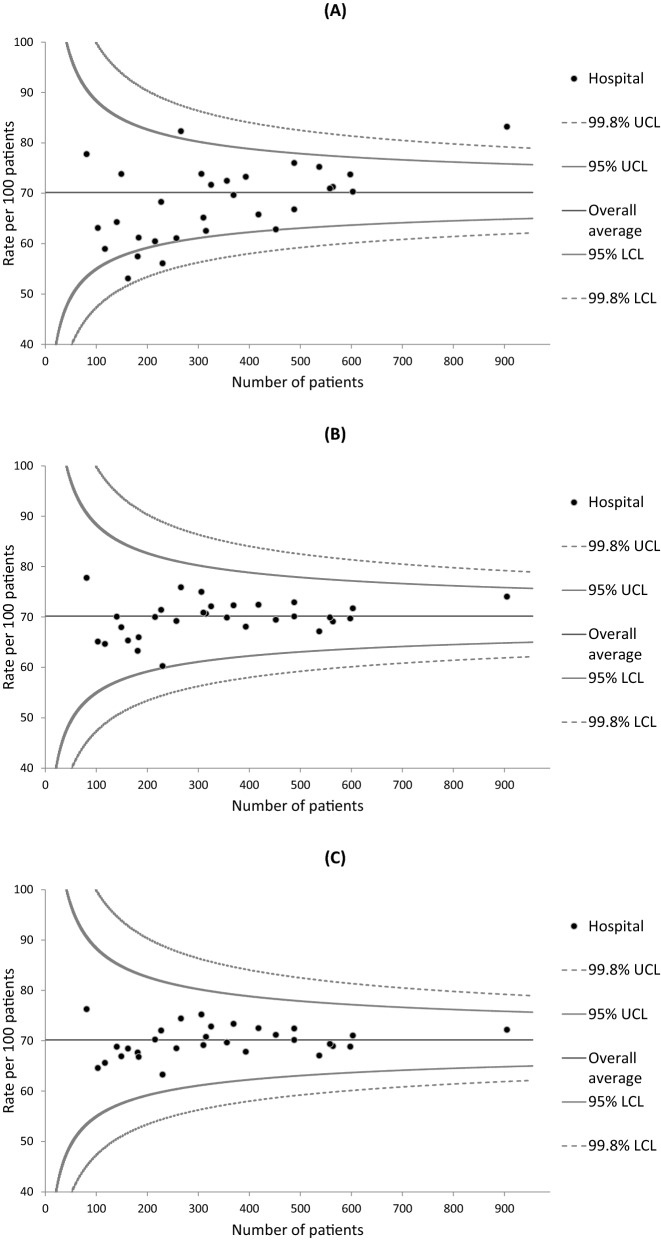
Funnel plots of 3-year survival and the number of gastric cancer patients in each hospital. (**A**) Unadjusted survival; (**B**) Partially adjusted survival that controlled for age, sex, and cancer stage; and (**C**) Fully adjusted survival that controlled for comorbidities, activities of daily living, type of admission, smoking status, and body mass index in addition to the variables in the partially adjusted model. *LCL* lower control limit, *UCL* upper control limit.

Funnel plots describing the unadjusted, partially adjusted, and fully adjusted 3-year survival rates of gastric cancer for 30 hospitals are presented in Fig. 2. The unadjusted survival rates ranged from 53.0 to 83.2% among the hospitals. The funnel plot highlighted five hospitals with unadjusted survival rates below the lower control limit or above the upper control limit of 2 SDs (Fig. 2A). The unadjusted survival rates for the remaining 25 hospitals fell between these lower and upper control limits. After risk adjustment, however, the between-hospital variations in survival rates had shrunk. The partially adjusted and fully adjusted survival rates for all 30 hospitals fell between the lower and upper control limits of 2 SDs, with variations shrinking further in the latter according to visual inspection (Fig. 2B,C).

**Figure 3:**
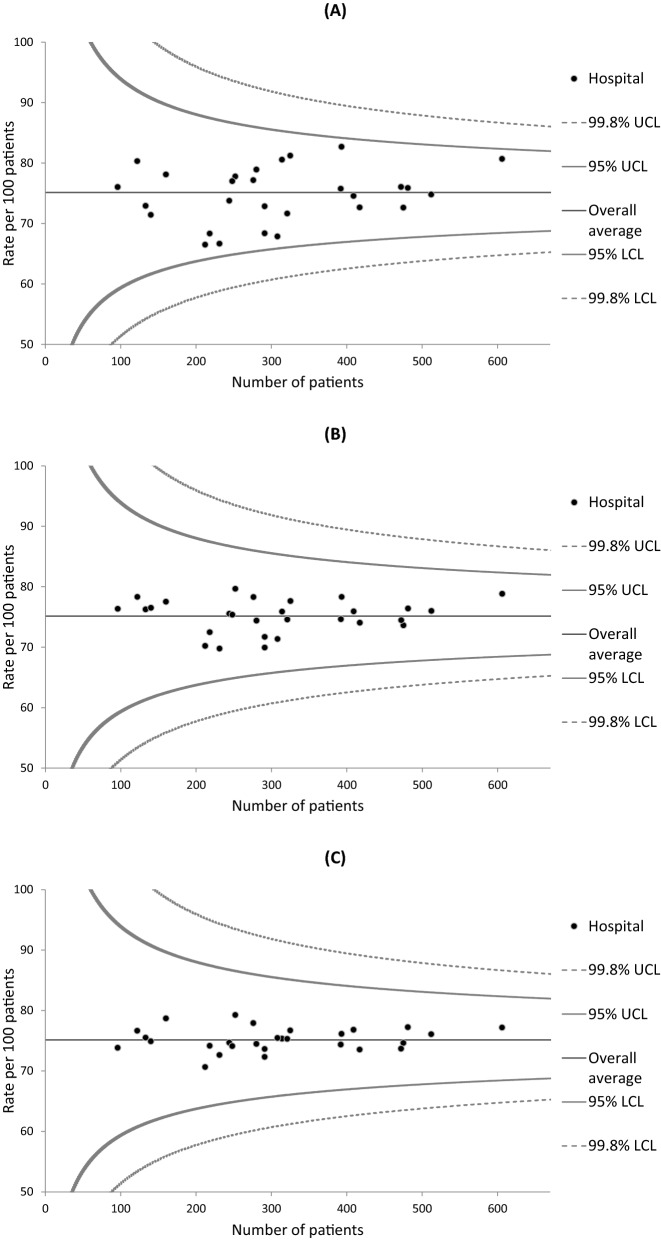
Funnel plots of 3-year survival and the number of colorectal cancer patients in each hospital. (**A**) Unadjusted survival; (**B**) Partially adjusted survival that controlled for age, sex, cancer stage, and tumor localization; and (**C**) Fully adjusted survival that controlled for comorbidities, activities of daily living, type of admission, smoking status, and body mass index in addition to the variables in the partially adjusted model. *LCL* lower control limit, *UCL* upper control limit.

Funnel plots describing the unadjusted, partially adjusted, and fully adjusted 3-year survival rates of colorectal cancer for 30 hospitals are presented in Fig. 3. The unadjusted survival rates ranged from 66.5 to 82.7% among the hospitals. The survival rates for all 30 hospitals lay between the lower and upper control limits of 2 SDs even before risk adjustment. However, the adjustments reduced the variations according to visual inspection.

**Figure 4:**
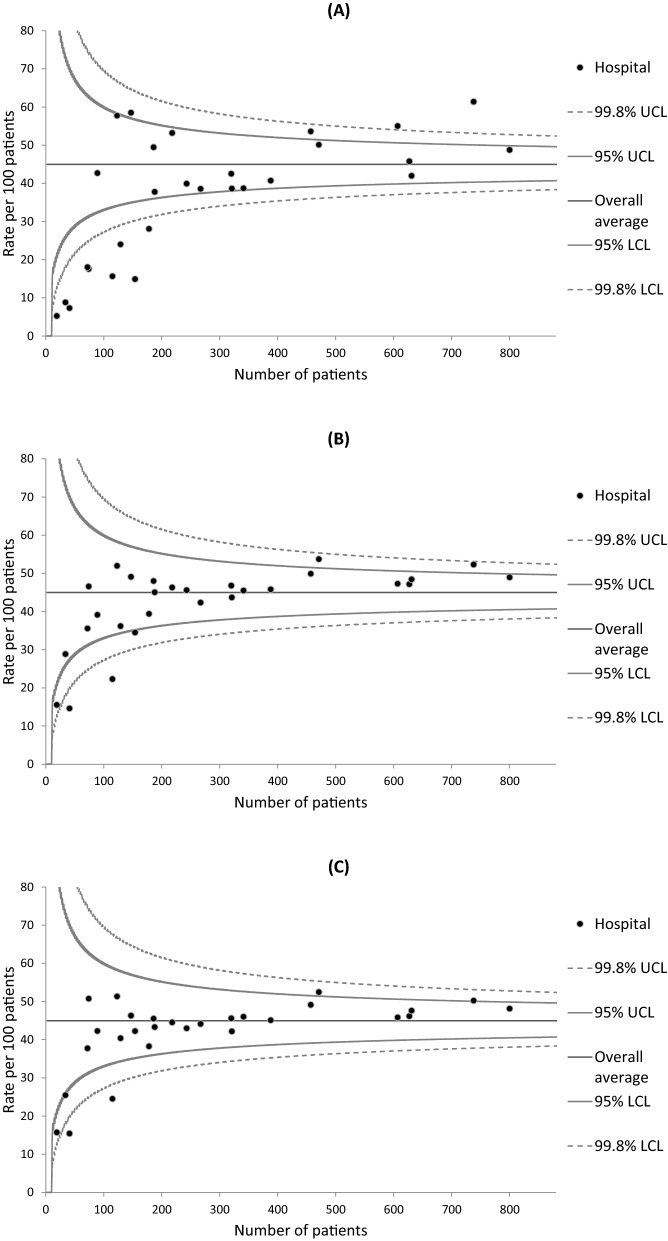
Funnel plots of 3-year survival and the number of lung cancer patients in each hospital. (**A**) Unadjusted survival; (**B**) Partially adjusted survival that controlled for age, sex, cancer stage, and histology; and (**C**) Fully adjusted survival that controlled for comorbidities, activities of daily living, type of admission, smoking status, and body mass index in addition to the variables in the partially adjusted model. *LCL* lower control limit, *UCL* upper control limit.

Funnel plots describing the unadjusted, partially adjusted, and fully adjusted 3-year survival rates of lung cancer for 28 hospitals are presented in Fig. 4. The unadjusted survival rates ranged from 5.3 to 61.4% among the hospitals. The funnel plot detected 13 hospitals with unadjusted survival rates below the lower control limit or above the upper control limit of 2 SDs (Fig. 4A). The unadjusted survival rates for the remaining 15 hospitals fell between these lower and upper control limits. In Fig. 4B, the partially adjusted survival rates for six hospitals remained below the lower control limit or above the upper control limit of 2 SDs. In Fig. 4C, the funnel plot identified two hospitals with fully adjusted survival rates lying below the lower control limit of 3 SDs (“alarm” line), two hospitals with fully adjusted survival rates lying below the lower control limit of 2 SDs (“alert” line), and two hospitals with fully adjusted survival rates lying above the upper control limit of 2 SDs. The fully adjusted survival rates for the remaining 22 hospitals fell between these lower and upper control limits, with variations shrinking further according to visual inspection when compared with the partially adjusted survival rates.

### Subgroup analyses

The study population for the subgroup analyses of stage I cancer patients comprised 6400 gastric cancer patients, 2536 colorectal cancer patients, and 2548 lung cancer patients. As shown in Supplementary Table S2, restricting the study population to patients with stage I cancer did not markedly affect the results of the multivariable analyses on the associations of the potential prognostic factors with 3-year survival after multiple imputations. Funnel plots describing the 3-year survival rates of gastric cancer for 30 hospitals, colorectal cancer for 30 hospitals, and lung cancer for 22 hospitals are presented in Supplementary Figs. S1, S2, and S3, respectively. The survival rates of gastric cancer and colorectal cancer for all hospitals lay between the lower and upper control limits of 2 SDs before and after risk adjustment. The funnel plots for lung cancer indicated that although one hospital had an unadjusted rate below the lower control limit of 2 SDs, its partially and fully adjusted survival rates fell between the lower and upper control limits.

## Discussion

Using a dataset that linked cancer registry data and administrative data, this study examined the variations in cancer survival rates among Japanese cancer care hospitals over the course of 3 years irrespective of treatment modality. We observed wide between-hospital variations in the unadjusted survival rates of gastric and lung cancer patients, which were reduced after adjusting for case mix. Interestingly, there were no substantial between-hospital variations in survival among colorectal cancer patients even before risk adjustment. A possible explanation for this finding may be that prognostic factors such as age, sex, and cancer stage were weaker in predicting the survival duration for colorectal cancer than the other two cancer types, which in turn had little impact on the between-hospital differences. Another explanation may be that colorectal cancer patients have modest healthcare seeking behavior, which suggests that the equalization of healthcare services for this cancer type has been virtually attained^42^. It was notable that only a small proportion of patients had missing data (“unknown” categories) for the potential prognostic factors, and these were included in the risk adjustment models to provide a more valid investigation of institutional variations. A strength of our study was the examination of survival over several years. As postoperative death has become a relatively rare event, the appraisal of cancer care services requires the inclusion of long-term oncological outcomes^29,31,32^.

Through a visual comparison of the partially and fully adjusted survival rates in the funnel plots, we found that several demographic factors from the administrative data could partly explain the between-hospital variations. These results underscored the importance of record linkage for better risk adjustment because both administrative and registry data contribute unique prognostic information. In fact, numerous cancer-related studies on health services research have similarly used record-linked databases such as the SEER-Medicare Linked Database^5–7,12,14–20,22–24,32^.

The observed variations in survival rates that persisted even after adjusting for case mix may be explained by differences in provided care. While this study identified hospitals with outlying 3-year survival rates, the mechanisms underlying these variations remain unclear. One explanation is that hospitals with a higher survival rate are more adept at providing higher-quality care^7,8^. If this is true, an effective strategy for reducing variations in outcomes would be to learn best practice from the hospitals with the best outcomes and to spread its adoption^2,3^. Other potential factors may include staffing, surgeon proficiency, perioperative management, multidisciplinary teams, palliative care, and patient safety culture^4^. In addition, hospital caseload has been correlated with outcomes in cancer patients^43^. Investigating and addressing the causes of the observed variations could improve the care and outcomes of the cancer population.

Caution is needed when considering how institutional variations in outcomes should be applied to improve the quality of care. First, the definition of outlying status is complicated. To simplify this definition, our approach to identifying statistical outliers on the basis of control limits using 2 SDs and 3 SDs followed previously published studies^23–29^. Second, hospitals may not necessarily be excellent or substandard performers for all process or outcome metrics despite being high or low outliers for the survival outcome. Performance appraisals of service quality in cancer treatment is multifaceted and dependent on the viewpoints of the stakeholders concerned. Third, a “snapshot” of performance cannot be safely interpreted in isolation. It would be erroneous to form any firm conclusions based on a single data point where a hospital happens to be an outlier, and more prudent to identify persistent outliers in time series analyses of quality metrics. Fourth, the institutional variations can also be explained by data quality. Not all prognostic factors could be quantified in this study. Specifically, hospitals that treat more patients with severe diseases are more likely to have a higher premature mortality rate, and this disadvantage was not directly discernible from our data sources.

Nevertheless, the between-hospital variations in survival should not be ignored. These variations suggest that there may be considerable scope for quality improvement^9^. Efforts should be made to elucidate why outlier hospitals had significantly better or worse values than other hospitals. Our findings provide a basis for future in-depth research and clinical audits that aim to eliminate disparities and improve the quality of clinical practice. Ultimately, the identification of variations is only beneficial if it can contribute to quality improvement initiatives^2,3^.

The prognostic factors of overall survival identified in this study were generally concordant with those identified in previous reports^14–16,35–39^. The associations of comorbidities and functional disability with premature mortality could be explained by compromised cancer treatment plans and poorer health conditions^14,15^. Furthermore, the poorer survival associated with emergency admissions may be indicative of higher complication rates and biologically aggressive tumors in these patients^38,39^. The association between low BMI and poorer survival could be attributed to reduced dietary intake, sarcopenia, and cachexia^16^.

## Limitations

This study has several limitations. First, and most importantly, the risk adjustment models lacked detailed oncological information such as genetic factors. As stated above, case mix factors that could not be accounted for in the dataset may influence outcomes. Nevertheless, risk adjustments were conducted for many of the important factors known to influence survival. Second, we only analyzed data from designated cancer care hospitals. For this reason, our study population may not be representative of the whole population in the study region, and could be vulnerable to selection bias. The inclusion of all hospitals in the region may produce even larger variations than those observed in our analysis.

## Conclusions

We identified between-hospital variations in 3-year survival for patients with gastric, colorectal, and lung cancer diagnosed between 2013 and 2015. Benchmarking institutional oncological performance is complex and should not be generalized from a single outcome metric. However, the elucidation of these variations can support future studies that explore the underlying clinical mechanisms for quality improvement initiatives, which will contribute to the improvement of treatment quality and outcomes.

## Supplementary Information

Supplementary Information.

## Data Availability

The datasets analyzed during the current study are available from the corresponding author on reasonable request.

## References

[1] GBD 2016 Healthcare Access and Quality Collaborators (2018). Measuring performance on the Healthcare Access and Quality Index for 195 countries and territories and selected subnational locations: A systematic analysis from the Global Burden of Disease Study 2016. Lancet.

[2] Dickman SL, Himmelstein DU, Woolhandler S (2017). Inequality and the health-care system in the USA. Lancet.

[3] Stephens TJ (2020). Hospital-level evaluation of the effect of a national quality improvement programme: Time-series analysis of registry data. BMJ Qual. Saf..

[4] du Bois A, Rochon J, Pfisterer J, Hoskins WJ (2009). Variations in institutional infrastructure, physician specialization and experience, and outcome in ovarian cancer: A systematic review. Gynecol. Oncol..

[5] Haneuse S, Dominici F, Normand SL, Schrag D (2018). Assessment of between-hospital variation in readmission and mortality after cancer surgical procedures. JAMA Netw. Open.

[6] Fong ZV (2020). Variation in long-term oncologic outcomes by type of cancer center accreditation: An analysis of a SEER-Medicare population with pancreatic cancer. Am. J. Surg..

[7] Okuyama A, Higashi T (2018). Patterns of cancer treatment in different age groups in Japan: An analysis of hospital-based cancer registry data, 2012–2015. Jpn. J. Clin. Oncol..

[8] Yasunaga H, Hashimoto H, Horiguchi H, Miyata H, Matsuda S (2012). Variation in cancer surgical outcomes associated with physician and nurse staffing: A retrospective observational study using the Japanese Diagnosis Procedure Combination Database. BMC Health Serv. Res..

[9] Portela MC, Pronovost PJ, Woodcock T, Carter P, Dixon-Woods M (2015). How to study improvement interventions: A brief overview of possible study types. BMJ Qual. Saf..

[10] Remark R (2015). The non-small cell lung cancer immune contexture: A major determinant of tumor characteristics and patient outcome. Am. J. Respir. Crit. Care Med..

[11] Iezzoni LI (1995). Risk adjustment for medical effectiveness research: An overview of conceptual and methodological considerations. J. Investig. Med..

[12] Warren JL, Klabunde CN, Schrag D, Bach PB, Riley GF (2002). Overview of the SEER-Medicare data: Content, research applications, and generalizability to the United States elderly population. Med. Care.

[13] Hayashida K, Murakami G, Matsuda S, Fushimi K (2021). History and profile of Diagnosis Procedure Combination (DPC): Development of a real data collection system for acute inpatient care in Japan. J. Epidemiol..

[14] Morishima T (2019). Impact of comorbidities on survival in gastric, colorectal, and lung cancer patients. J. Epidemiol..

[15] Morishima T (2021). Barthel Index-based functional status as a prognostic factor in young and middle-aged adults with newly diagnosed gastric, colorectal and lung cancer: A multicentre retrospective cohort study. BMJ Open.

[16] Morishima T, Sato A, Nakata K, Miyashiro I (2020). Geriatric assessment domains to predict overall survival in older cancer patients: An analysis of functional status, comorbidities, and nutritional status as prognostic factors. Cancer Med..

[17] Nishikawa T (2021). Multicentre cohort study of the impact of percutaneous coronary intervention on patients with concurrent cancer and ischaemic heart disease. BMC Cardiovasc. Disord..

[18] Fuji S (2021). Analysis of real-world data in patients with relapsed/refractory diffuse large B cell lymphoma who received salvage chemotherapy in the rituximab era. Ann. Hematol..

[19] Kida S (2021). Comparison of CHOP with THP-COP for peripheral T-cell lymphoma-not otherwise specified and angioimmunoblastic T-cell lymphoma: A retrospective analysis using data from the population-based Osaka Cancer Registry. Int. J. Hematol..

[20] Kida N, Morishima T, Tsubakihara Y, Miyashiro I (2022). Stage at diagnosis and prognosis of colorectal, stomach, lung, liver, kidney, and bladder cancers in dialysis patients: A multicenter retrospective study using cancer registry data and administrative data. Nephron.

[21] Wong SL (2015). Variation in hospital mortality rates with inpatient cancer surgery. Ann. Surg..

[22] Osler M (2011). Hospital variation in 30-day mortality after colorectal cancer surgery in Denmark: The contribution of hospital volume and patient characteristics. Ann. Surg..

[23] Jorgensen ML, Young JM, Dobbins TA, Solomon MJ (2014). Predictors of variation in colorectal cancer care and outcomes in New South Wales: A population-based health data linkage study. Med. J. Aust..

[24] Morris EJ (2011). Thirty-day postoperative mortality after colorectal cancer surgery in England. Gut.

[25] Damhuis RA, Maat AP, Plaisier PW (2015). Performance indicators for lung cancer surgery in the Netherlands. Eur. J. Cardiothorac. Surg..

[26] O'Kane R, Mathew R, Kenny T, Stiller C, Chumas P (2013). United Kingdom 30-day mortality rates after surgery for pediatric central nervous system tumors. J. Neurosurg. Pediatr..

[27] Teloken PE, Spilsbury K, Platell C, Committee BO (2016). Analysis of mortality in colorectal surgery in the Bi-National Colorectal Cancer Audit. ANZ J. Surg..

[28] De Witt Hamer PC (2019). Between-hospital variation in mortality and survival after glioblastoma surgery in the Dutch Quality Registry for Neuro Surgery. J. Neurooncol..

[29] Almoudaris AM (2013). Single measures of performance do not reflect overall institutional quality in colorectal cancer surgery. Gut.

[30] Wallington M (2016). 30-day mortality after systemic anticancer treatment for breast and lung cancer in England: A population-based, observational study. Lancet Oncol..

[31] Solheim O, Jakola AS, Gulati S, Johannesen TB (2012). Incidence and causes of perioperative mortality after primary surgery for intracranial tumors: A national, population-based study. J. Neurosurg..

[32] Pfister DG (2015). Risk adjusting survival outcomes in hospitals that treat patients with cancer without information on cancer stage. JAMA Oncol..

[33] Albert JM, Das P (2012). Quality assessment in oncology. Int. J. Radiat. Oncol. Biol. Phys..

[34] Lee GH (2015). Is right-sided colon cancer different to left-sided colorectal cancer? A systematic review. Eur. J. Surg. Oncol..

[35] Asamura H (2008). A Japanese Lung Cancer Registry study: Prognosis of 13,010 resected lung cancers. J. Thorac. Oncol..

[36] Quan H (2011). Updating and validating the Charlson comorbidity index and score for risk adjustment in hospital discharge abstracts using data from 6 countries. Am. J. Epidemiol..

[37] Quan H (2005). Coding algorithms for defining comorbidities in ICD-9-CM and ICD-10 administrative data. Med. Care.

[38] Murchie P (2014). Time from first presentation in primary care to treatment of symptomatic colorectal cancer: Effect on disease stage and survival. Br. J. Cancer.

[39] Amri R, Bordeianou LG, Sylla P, Berger DL (2015). Colon cancer surgery following emergency presentation: Effects on admission and stage-adjusted outcomes. Am. J. Surg..

[40] White IR, Royston P, Wood AM (2011). Multiple imputation using chained equations: Issues and guidance for practice. Stat. Med..

[41] Spiegelhalter DJ (2005). Funnel plots for comparing institutional performance. Stat. Med..

[42] Tanaka H, Ishikawa KB, Katanoda K (2018). Geographic access to cancer treatment in Japan: Results from a combined dataset of the Patient Survey and the Survey of Medical Institutions in 2011. J. Epidemiol..

[43] Pieper D, Mathes T, Neugebauer E, Eikermann M (2013). State of evidence on the relationship between high-volume hospitals and outcomes in surgery: A systematic review of systematic reviews. J. Am. Coll. Surg..

