# Supplementary material for: Between-hospital variations in 3-year survival among patients with newly diagnosed gastric, colorectal, and lung cancer
Source: Sci Rep. 2022 May 3;12:7134. doi: 10.1038/s41598-022-11225-5 (PMC9065118; doi:10.1038/s41598-022-11225-5)

Supplementary Information for

Between-hospital variations in three-year survival among patients with newly diagnosed gastric, colorectal, and lung cancer

Toshitaka Morishima, Sumiyo Okawa, Shihoko Koyama,
Kayo Nakata, Takahiro Tabuchi, Isao Miyashiro

Contents

- **Supplementary Table S1.** Association of prognostic factors with three-year overall survival in patients with gastric, colorectal, and lung cancer using complete-case data.

- **Supplementary Table S2.** Association of prognostic factors with three-year overall survival in patients with stage I gastric, stage I colorectal, and stage I lung cancer using data from multiple imputation models.

- **Supplementary Figure S1.** Funnel plots of three-year survival and the number of stage I gastric cancer patients in each hospital. (A) Unadjusted survival; (B) Partially adjusted survival that controlled for age and sex; and (C) Fully adjusted survival that controlled for comorbidities, activities of daily living, type of admission, smoking status, and body mass index in addition to the variables in the partially adjusted model. LCL, lower control limit; UCL, upper control limit.

- **Supplementary Figure S2.** Funnel plots of three-year survival and the number of stage I colorectal cancer patients in each hospital. (A) Unadjusted survival; (B) Partially adjusted survival that controlled for age, sex, and tumor localization; and (C) Fully adjusted survival that controlled for comorbidities, activities of daily living, type of admission, smoking status, and body mass index in addition to the variables in the partially adjusted model. LCL, lower control limit; UCL, upper control limit.

- **Supplementary Figure S3.** Funnel plots of three-year survival and the number of stage I lung cancer patients in each hospital. (A) Unadjusted survival; (B) Partially adjusted survival that controlled for age, sex, and histology; and (C) Fully adjusted survival that controlled for comorbidities, activities of daily living, type of admission, smoking status, and body mass index in addition to the variables in the partially adjusted model. LCL, lower control limit; UCL, upper control limit.

Supplementary Table S1 Association of prognostic factors with three-year overall survival in patients with gastric, colorectal, and lung cancer using complete-case data

	Gastric cancer (n=9814)		Colorectal cancer (n=8829)		Lung cancer (n=7612)	
	Adjusted odds ratio	*P* value	Adjusted odds ratio	*P* value	Adjusted odds ratio	*P* value
Age (years; Ref = 18–64)						
65–69	0.83 (0.68–1.02)	0.076	0.94 (0.77–1.15)	0.545	0.68 (0.57–0.82)	<0.001
70–74	0.63 (0.51–0.76)	<0.001	0.84 (0.69–1.01)	0.064	0.55 (0.46–0.65)	<0.001
75–79	0.44 (0.36–0.53)	<0.001	0.53 (0.43–0.64)	<0.001	0.33 (0.28–0.40)	<0.001
80–99	0.25 (0.21–0.31)	<0.001	0.35 (0.29–0.42)	<0.001	0.18 (0.15–0.23)	<0.001
Sex (Ref = female)						
Male	0.68 (0.58–0.80)	<0.001	0.78 (0.68–0.89)	<0.001	0.54 (0.46–0.63)	<0.001
Stage (Ref = I)						
II	0.37 (0.30–0.45)	<0.001	0.94 (0.77–1.14)	0.508	0.31 (0.26–0.38)	<0.001
III	0.11 (0.09–0.12)	<0.001	0.43 (0.36–0.51)	<0.001	0.12 (0.10–0.14)	<0.001
IV	0.01 (0.01–0.01)	<0.001	0.04 (0.03–0.05)	<0.001	0.03 (0.03–0.04)	<0.001
Tumor localization (Ref = left-sided)						
Right-sided	–		0.93 (0.82–1.06)	0.258	–	
Histology (Ref = NSCLC)						
SCLC	–		–		0.39 (0.31–0.49)	<0.001
Comorbidity (Ref = no comorbidity)						
Moderate comorbidities	0.65 (0.57–0.75)	<0.001	0.63 (0.55–0.72)	<0.001	0.88 (0.76–1.01)	0.060
Severe comorbidities	0.34 (0.25–0.46)	<0.001	0.34 (0.26–0.45)	<0.001	0.59 (0.45–0.79)	<0.001
ADL (Ref = no disability)						
Moderate disability	0.50 (0.40–0.63)	<0.001	0.49 (0.40–0.62)	<0.001	0.50 (0.40–0.63)	<0.001
Severe disability	0.20 (0.15–0.28)	<0.001	0.33 (0.26–0.41)	<0.001	0.24 (0.16–0.36)	<0.001
Type of admission (Ref = elective)						
Emergency	0.70 (0.55–0.89)	0.004	0.68 (0.58–0.81)	<0.001	0.52 (0.37–0.73)	<0.001
Smoking status (Ref = never smoker)						
Current or ex-smoker	0.91 (0.79–1.05)	0.181	0.86 (0.75–0.99)	0.035	0.48 (0.41–0.57)	<0.001
Body mass index (Ref = 18.5–24.9)						
<18.5	0.55 (0.46–0.66)	<0.001	0.66 (0.55–0.78)	<0.001	0.55 (0.46–0.67)	<0.001
25.0–29.9	1.25 (1.05–1.47)	0.010	1.39 (1.18–1.64)	<0.001	1.13 (0.97–1.33)	0.125
≥30.0	1.51 (0.96–2.36)	0.074	1.20 (0.82–1.75)	0.351	1.01 (0.67–1.52)	0.964

ADL, activities of daily living; NSCLC, non-small cell lung cancer; Ref, reference; SCLC, small cell lung cancer.

Supplementary Table S2 Association of prognostic factors with three-year overall survival in patients with stage I gastric, stage I colorectal, and stage I lung cancer using data from multiple imputation models

	**Gastric cancer (n=6400)**		**Colorectal cancer (n=2536)**		**Lung cancer (n=2548)**	
	Adjusted odds ratio	*P* value	Adjusted odds ratio	*P* value	Adjusted odds ratio	*P* value
Age (years; Ref = 18–64)						
65–69	0.50 (0.34–0.75)	<0.001	0.67 (0.39–1.15)	0.145	0.52 (0.33–0.82)	0.005
70–74	0.35 (0.24–0.50)	<0.001	0.50 (0.30–0.83)	0.007	0.47 (0.31–0.71)	<0.001
75–79	0.24 (0.17–0.35)	<0.001	0.40 (0.24–0.66)	<0.001	0.34 (0.22–0.52)	<0.001
80–99	0.15 (0.10–0.21)	<0.001	0.23 (0.14–0.38)	<0.001	0.15 (0.09–0.23)	<0.001
Sex (Ref = female)						
Male	0.58 (0.46–0.74)	<0.001	0.58 (0.40–0.83)	0.003	0.67 (0.49–0.90)	0.009
Tumor localization (Ref = left-sided)						
Right-sided	–		1.42 (1.02–1.97)	0.036	–	
Histology (Ref = NSCLC)						
SCLC	–		–		0.25 (0.14–0.46)	<0.001
Comorbidity (Ref = no comorbidity)						
Moderate comorbidities	0.47 (0.39–0.57)	<0.001	0.51 (0.37–0.71)	<0.001	0.71 (0.55–0.91)	0.007
Severe comorbidities	0.30 (0.21–0.43)	<0.001	0.23 (0.13–0.39)	<0.001	0.48 (0.31–0.74)	<0.001
ADL (Ref = no disability)						
Moderate disability	0.43 (0.31–0.58)	<0.001	0.36 (0.22–0.59)	<0.001	0.46 (0.31–0.69)	<0.001
Severe disability	0.16 (0.11–0.23)	<0.001	0.30 (0.16–0.53)	<0.001	0.06 (0.03–0.13)	<0.001
Type of admission (Ref = elective)						
Emergency	0.44 (0.28–0.68)	<0.001	0.34 (0.19–0.62)	<0.001	0.55 (0.19–1.53)	0.250
Smoking status (Ref = never smoker)						
Current or ex-smoker	0.76 (0.61–0.93)	0.010	0.83 (0.59–1.16)	0.276	0.34 (0.24–0.48)	<0.001
Body mass index (Ref = 18.5–24.9)						
<18.5	0.43 (0.34–0.56)	<0.001	0.41 (0.27–0.63)	<0.001	0.46 (0.32–0.64)	<0.001
25.0–29.9	1.40 (1.09–1.79)	0.008	1.43 (0.96–2.12)	0.081	1.02 (0.76–1.38)	0.900
≥30.0	1.24 (0.65–2.37)	0.521	1.90 (0.62–5.77)	0.258	0.79 (0.37–1.66)	0.535

ADL, activities of daily living; NSCLC, non-small cell lung cancer; Ref, reference; SCLC, small cell lung cancer.

Supplementary Figure S1

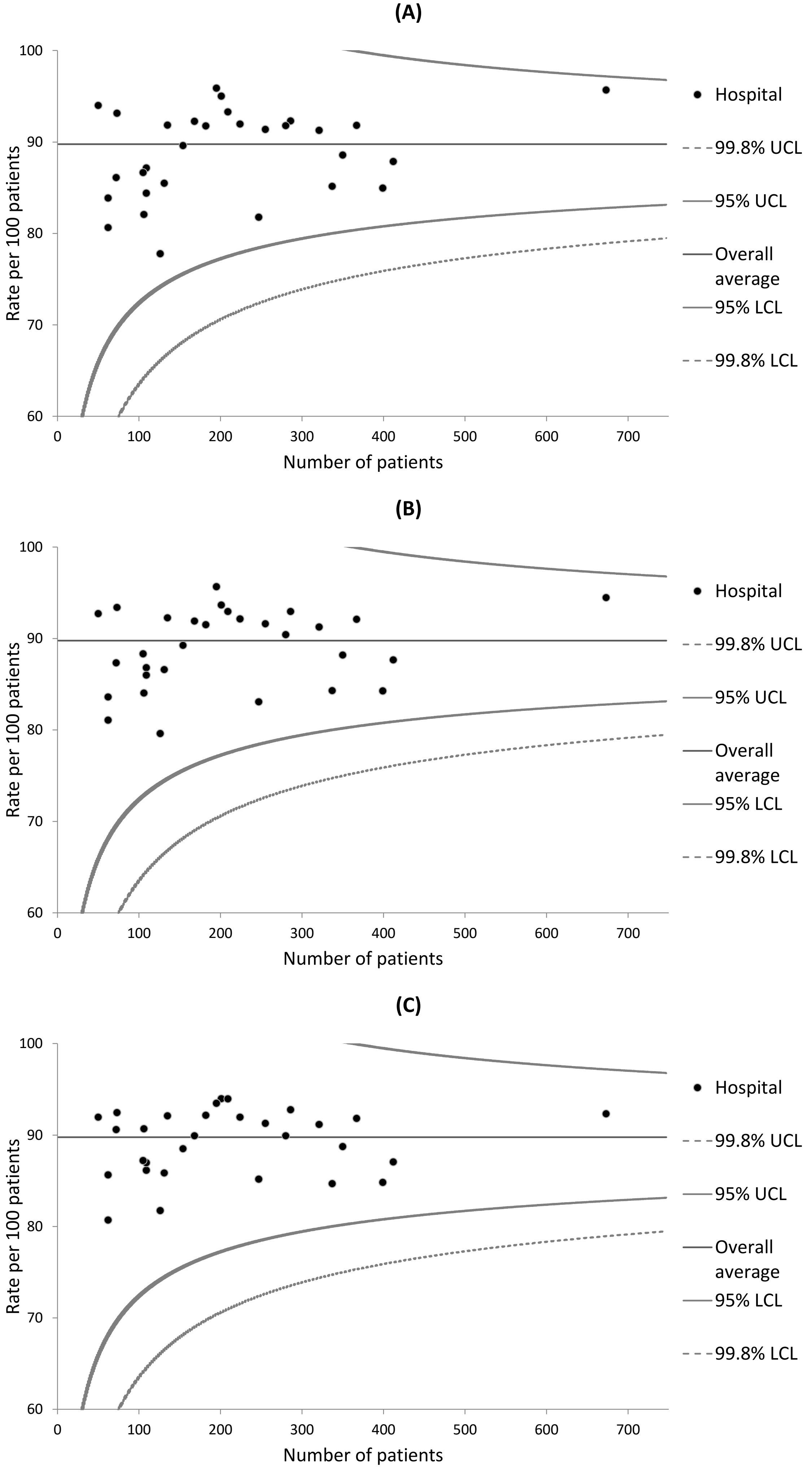

Supplementary Figure S2

(A)

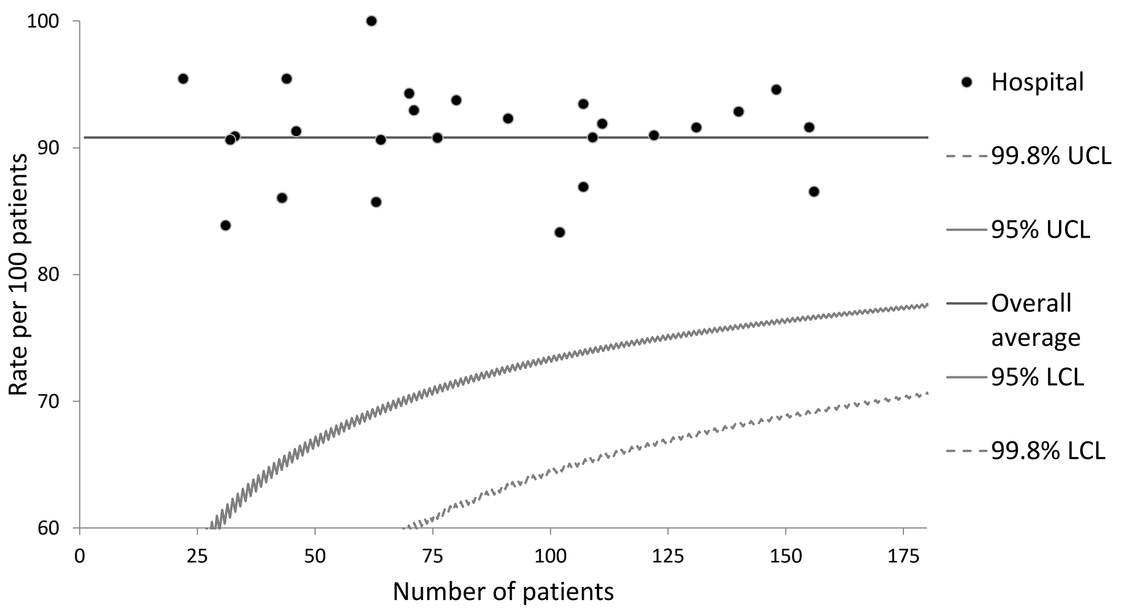

(B)

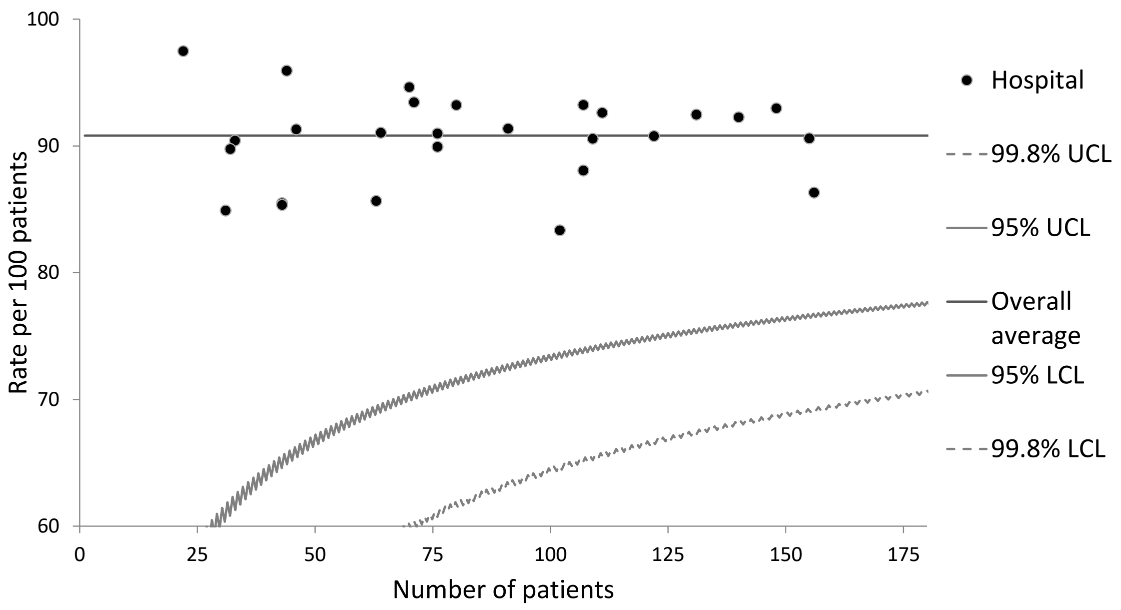

(C)

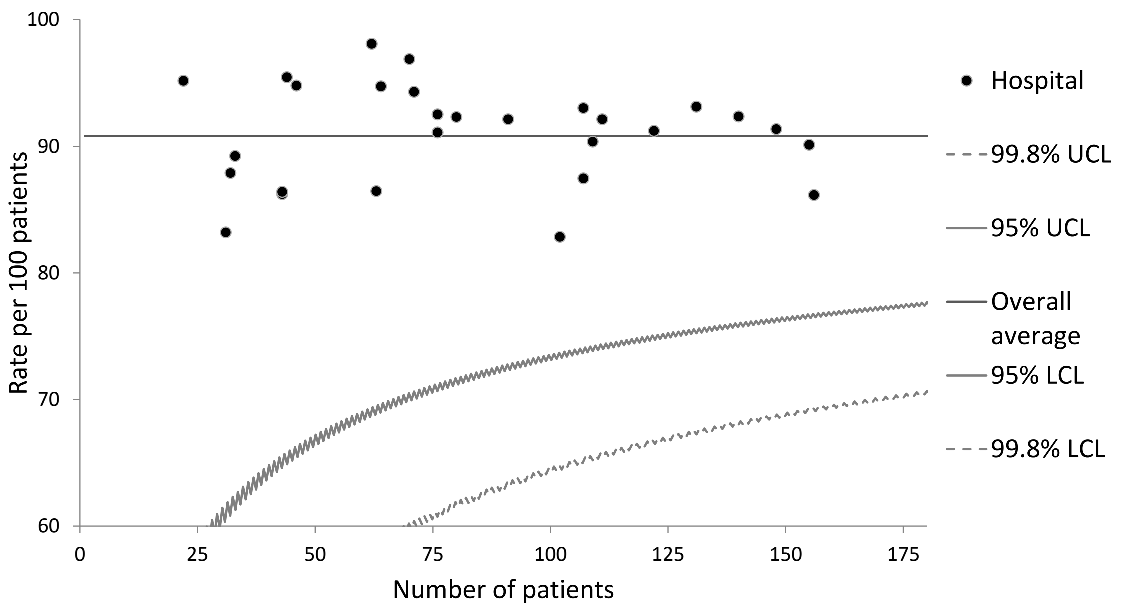

Supplementary Figure S3

(A)

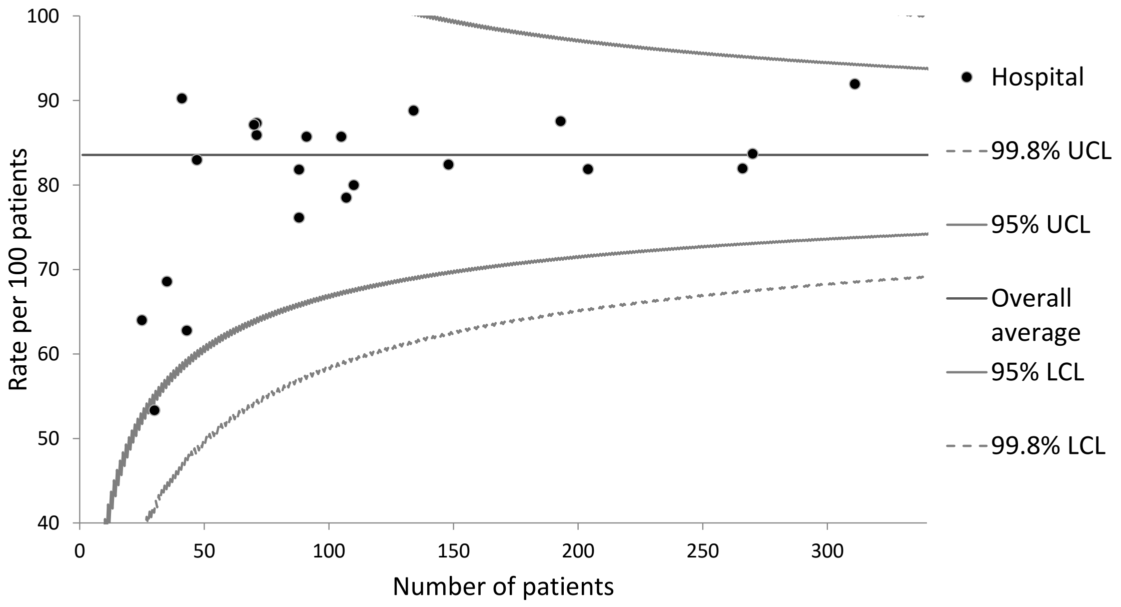

(B)

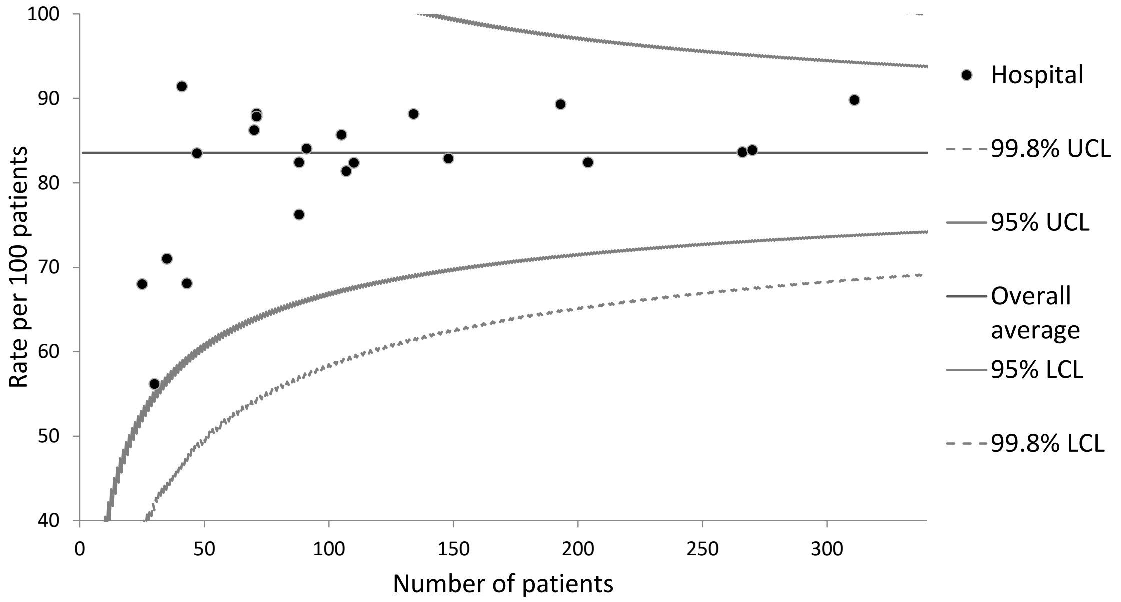

(C)

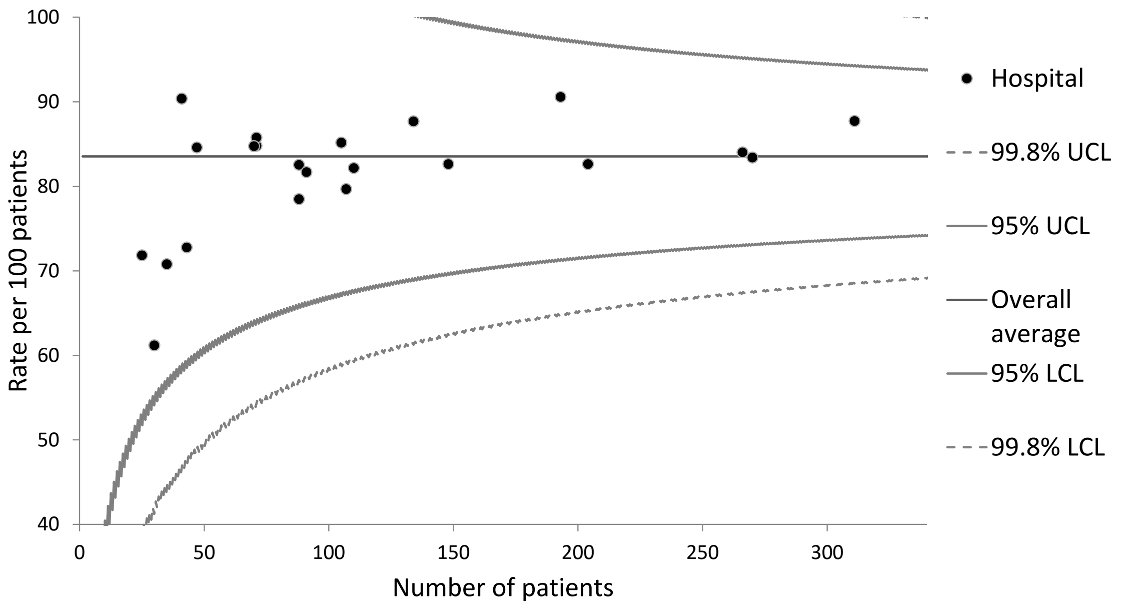

Supplement: Supplementary file 1 — Supplementary Information. [file 41598_2022_11225_MOESM1_ESM.pdf]
